# What are the effects of varenicline compared with nicotine replacement therapy on long-term smoking cessation and clinically important outcomes? Protocol for a prospective cohort study

**DOI:** 10.1136/bmjopen-2015-009665

**Published:** 2015-11-05

**Authors:** Neil M Davies, Gemma Taylor, Amy E Taylor, Kyla H Thomas, Frank Windmeijer, Richard M Martin, Marcus R Munafò

**Affiliations:** 1 Medical Research Council Integrative Epidemiology Unit, University of Bristol, Bristol, UK; 2 School of Social and Community Medicine, University of Bristol, Bristol, UK; 3 School of Experimental Psychology, University of Bristol, Bristol, UK; 4 Department of Economics, University of Bristol, Bristol, UK

**Keywords:** STATISTICS & RESEARCH METHODS, EPIDEMIOLOGY, INTERNAL MEDICINE

## Abstract

**Introduction:**

Smoking is a major avoidable cause of ill-health and premature death. Treatments that help patients successfully quit smoking have an important effect on health and life expectancy. Varenicline is a medication that can help smokers successfully quit smoking. However, there are concerns that it may cause adverse effects, such as increase in the occurrence of depression, self-harm and suicide and cardiovascular disease. In this study we aim to examine the effects of varenicline versus other smoking cessation pharmacotherapies on smoking cessation, health service use, all-cause and cause-specific mortality and physical and mental health conditions.

**Methods:**

In this project we will investigate the effects of varenicline compared to nicotine replacement therapies on: (1) long-term smoking cessation and whether these effects differ by area level deprivation; and (2) the following clinically-important outcomes: rate of general practice and hospital attendance; all-cause mortality and death due to diseases of the respiratory system and cardiovascular disease; and a primary care diagnosis of respiratory illness, myocardial infarction or depression and anxiety. The study is based on a cohort of patients prescribed these smoking cessation medications from the Clinical Practice Research Datalink (CPRD). We will use three methods to overcome confounding: multivariable adjusted Cox regression, propensity score matched Cox regression, and instrumental variable regression. The total expected sample size for analysis will be at least 180 000. Follow-up will end with the earliest of either an ‘event’ or censoring due to the end of registration or death.

**Ethics and dissemination:**

Ethics approval was not required for this study. This project has been approved by the CPRD's Independent Scientific Advisory Committee (ISAC). We will disseminate our findings via publications in international peer-reviewed journals and presentations at international conferences.

**Strengths and limitations of this study**

- We will use data from a large sample of patients prescribed smoking cessation treatment in UK general practices. This means we will have substantial power to detect even relatively small effects, or effects on rare outcomes.
- We will use three statistical approaches to overcome confounding. These approaches depend on distinct assumptions. Triangulating across different methods will help provide more robust evidence about the effects of these medications.
- Our study will use observational data and so results can suffer from unobserved confounding. We will report in detail the confounding structure, and detail the methods we used to overcome this limitation.
- The outcomes used in this study will be defined using diagnoses and interactions that occur as part of the patients’ routine care. Different general practices may use a range of Read codes to record diagnoses. We will mitigate this limitation by using validated code lists, where available, to ensure the algorithms we use accurately capture the diagnoses and events of interest.

## Introduction

Smoking is the major cause of preventable morbidity and mortality in the UK and internationally.^1^ ^2^ Smoking is also the principal cause of health inequalities and is responsible for most of the difference in healthy life-expectancy between the richest and poorest in our society^3^ and those with and without mental health problems.^4^ ^5^ Smoking-related illnesses are estimated to cost the NHS approximately £5billion per year.^6^ Varenicline has been shown to be the most clinically effective smoking cessation medicine for short-term abstinence in randomised controlled trials (RCTs).^7^ However, there is relatively little evidence for its long-term effectiveness and impact on clinical outcomes.

Concerns have been raised that varenicline may be associated with a higher risk of adverse events, including suicide and self-harm and cardiovascular events, than other smoking cessation interventions.^8–11^ In 2009, the US Food and Drug Administration (FDA) mandated that varenicline carry a black box warning (the agency's strongest safety warning) highlighting the increased risk of suicidal ideation and depression in patients prescribed varenicline. This was based on spontaneous reports to the FDA Adverse Events Reporting (FDA AERS) database.^12^ These warnings are meant to indicate causal effects of pharmaceuticals. However, there are an increasing number of experimental and observational studies that suggest there is little difference in risk of adverse neuropsychiatric effects of varenicline compared to nicotine replacement products.^11^ ^13^ In October 2014, the FDA decided that the black box warning on varenicline should remain; it is expected that this guidance will be updated after publication of the results of the EAGLES randomised trial in late 2015.^14^ ^15^

Much of the evidence about the potential adverse effects of varenicline comes from observational studies, which are prone to confounding. We will add to the evidence base about the possible adverse and beneficial effects of prescribing different smoking cessation medications using three statistical approaches to overcome confounding: multivariable adjusted regression, propensity score regression and instrumental variable analysis. Using these three approaches we aim to examine the effects of varenicline versus other smoking cessation pharmacotherapies on smoking cessation, health service use, all-cause and cause-specific mortality and physical and mental health conditions. Follow-up will end with the earliest of either an ‘event’ or censoring due to the end of registration or death. We will not investigate bupropion because it is rarely prescribed and systematic reviews have found that it is less effective than varenicline for smoking cessation.^7^

### Study aims

1. What is the effect of varenicline on smoking abstinence? We will investigate the effects of varenicline on smoking abstinence because existing evidence from RCTs typically only followed participants for 1 year, and are not informative about long-term outcomes.
2. What are the effects of varenicline on NHS service use? We will investigate the effects of varenicline prescriptions on all-cause primary and secondary care utilisation because smoking increases morbidity and imposes major costs on the healthcare services.^16^
3. What are the effects of varenicline on all-cause and cause-specific mortality? We will investigate the incidence illnesses in the Clinical Practice Research Database (CPRD) using primary care diagnoses, admission to secondary care using ICD-10 codes, and Office of National Statistics (ONS) mortality records to maximise the number of events detected.
4. What are the effects of varenicline on common physical and mental health conditions? We will investigate the effects of varenicline on rates of cardiovascular outcomes and respiratory illnesses, as previous research has suggested that patients prescribed varenicline may have different rates of these outcomes.^9^ ^10^ Second, we will investigate the effects of varenicline on rates of depression and anxiety because there has been some reports that varenicline may reduce the risk of these outcomes.^13^
5. We will also examine differences in smoking cessation medication effectiveness by socioeconomic position. A recent systematic review reported that stop-smoking services may be helping to reduce inequalities in smoking prevalence by preferentially targeting smokers of lower socioeconomic position (SEP). Data from primary care records The Health Improvement Network (THIN) show that between 2008 and 2010, smokers in more deprived groups were more likely to receive smoking cessation interventions.^17^

## Methods and analysis

We will conduct a prospective cohort study of all patients prescribed varenicline or nicotine replacement products in the CPRD. Variables will be defined using ‘Read codes’ which are clinical encoding of patient phenomena, for example a patient's: occupation, demographic information, social circumstances, clinical symptoms and observations, laboratory tests and results, and diagnoses.

### Inclusion and exclusion criteria

We will include patients who were older than 18 and were prescribed medicines in British National Formulary (BNF) category 4.10.2 (Nicotine Dependence) from 1 September 2006, when varenicline was introduced to the UK, to the present. We will use patients prescribed other smoking cessation products (nicotine patches, gum, lozenges, and inhalers) as controls for patients prescribed varenicline. We will include patients whose records were classified as ‘acceptable’ by the CPRD from all ‘up to standard’ general practices (GPs) at least 18 months prior to date of entry of each cohort (1st March 2005). Up to standard GPs are practices that have submitted data that meets the CPRD quality control thresholds. Patient data are defined as ‘acceptable’ by the CPRD if they meet minimum quality control standards, for example their registration period with their GP is valid.

We will exclude patients who registered at a GP less than 365 days before the first recorded prescription, to allow for high-quality assessment of baseline data and possible confounders. Patients prescribed bupropion in the year before their index prescription of varenicline or nicotine replacement therapy will be excluded from the analysis. In the primary analysis we will exclude patients initially prescribed nicotine replacement therapies and varenicline together, although in our previous analysis this only occurred for 0.25% of all prescriptions.^13^

### Power calculations

The following power calculations are based on effect sizes and CIs observed in our previously published results, which used data from 110 000 individuals prescribed either varenicline or nicotine replacement therapy.^13^ Based on the rate of 18 000 new prescriptions per year observed in the CPRD from 2006 to 2011,^13^ we estimate that with a further 4 years of follow-up the number of patients prescribed either varenicline or nicotine replacement therapy will have increased by 72 000. Therefore the total expected sample size for analysis will be around 180 000.

In our previous analysis using CPRD data the age-adjusted and sex-adjusted HR for self-harm/suicide for varenicline versus nicotine replacement therapy at 9 months was 0.73 (95% CI 0.54 to 0.99); after adjusting for possible confounders this became 0.90 (95% CI: 0.66 to 1.22).^13^ A 70% increase in sample size would lead to a reduction of the SE by a factor of 1.3, reducing the breadth of the above CI in the adjusted analysis from 0.56 to 0.43.

Rare outcomes, such as self-harm and suicide were used in previous analyses; in this project we will have greater power to explore more common outcome measures. For example, in the previous analysis the 9-month age-adjusted and sex-adjusted HR for all-cause mortality 9 months after first prescription for varenicline versus nicotine replacement therapy was 0.43 (95% CI 0.35 to 0.53); after controlling for possible confounders this became 0.49 (95% CI 0.40 to 0.61). A 70% increase in sample size would lead to a reduction of the SE by a factor of 1.3, reducing the breadth of the above CI in the adjusted analysis from 0.21 to 0.16.

For the effects of varenicline versus nicotine replacement therapy on all-cause mortality, instrumental variable analysis found a risk difference of 0.7 (95% CI −3.3 to 4.7) per 1000 patients treated after 9 months. We estimate that a 70% increase in sample size would narrow the CIs from 8.0 to 6.2.

Using data from our previous project, within 2 years of first prescription, we found 2517 admissions for respiratory disease among 1374 patients; 3144 admissions for cardiovascular disease among 1022 patients; and 3277 admissions for depression or anxiety among 213 patients. This is more events than we found for suicide and self-harm in our previous study; therefore we believe that there will be enough events for this analysis.

To investigate differences in healthcare-seeking behaviour of smokers by SEP we will combine the sample used for the health outcomes described above with a sample of all other patients indicated as a current smoker after the 1 September 2006.

### Data collection and linkage

We will use linked Hospital Episodes Statistics (HES) and ONS mortality data to define frequency of GP and hospital attendance, frequency of all-cause and cause-specific hospitalisation and all-cause and cause-specific mortality. We will test these hypotheses using data from GP practices linked to external data sets only.

These are important hard outcomes for our study. We have already established that for certain outcomes, such as cause specific mortality the linked ONS data are more accurate.^18^ While it is possible to investigate these outcomes using CPRD data from GPs, the data are less precise and consistently recorded.^18^ Thus analyses using linked data are likely to be more precise. Furthermore, the linked data provide direct evidence about secondary care attendance of patients via the HES data. Again, while there are some data about referrals to secondary care in the main tables of the CPRD, the data are not as comprehensive as HES data. Our outcomes of interest occur after September 2006; therefore, we believe that the linked HES and ONS data will provide sufficient coverage for these outcomes.

### Data analysis

#### Exposure measures

First time users of the smoking cessation therapies (varenicline or nicotine replacement therapy) will be defined as people who received at least one prescription of the product after the 1 September 2006 but with no use of a related product during the 12 months before the index date (the first date on which a prescription was issued). Langley *et al*^19^ found the smoking cessation prescription data in the THIN database, which is closely related to the CPRD, to be highly comparable to national dispensing data. The prescriptions will be defined by the therapy file in the CPRD, which contains a list of all prescriptions issued to patients by their GP. Each therapy record records the date a prescription was issued, the quantity of drug prescribed and the dose.

The primary analysis will be limited to the first treatment episode. This is analogous to an intention-to-treat analysis in a RCT.^20^ This ensures that the target parameter estimated in the observational study will be comparable to the parameter estimated by a RCT. For example, if patients’ treatment adherence and duration is related to whether they experience adverse events, then definitions of exposure which are based on treatment adherence may provide unreliable evidence of the causal effect of the prescription on adverse outcomes. This analysis framework was described in Hernán *et al* (2008). The intuition is that most randomised trials recruit individuals and then split participants into different treatment arms. This means that comparison groups are implicitly from first treatment. The primary analysis in an RCT typically reports an intention to treat estimate, which is the difference in allocation between arms of the trial based on allocation, rather than the treatment the participant received. To obtain results that are comparable to a randomised trial Hernán argued that analysts need to construct cohorts that also follow-up patients from first treatment, rather using retrospectively defined exposures (eg, retrospectively defining exposure as ever exposed to varenicline).

To mimic an intention-to-treat analysis in an RCT, in our primary analysis patients who are initially prescribed nicotine replacement therapy, but later switch to varenicline, will be allocated to nicotine replacement therapy and vice-versa. We will use an intention-to-treat design for two reasons. First, while there are theoretical statistical models for estimating the effects of treatment switching such as marginal structural models, these methods require the strong assumption that there are no unmeasured confounders and typically require detailed data on time-varying confounders, which are unlikely to be available in the CPRD. Second, to our knowledge there are no instrumental variable methods to estimate the effects of switching treatment. However, we will investigate the number of participants who switch treatment as a sensitivity analysis.

#### Outcome measures

##### Outcome 1: Smoking abstinence

In the CPRD smoking status is indicated by whether the patient is a current, former or never smoker. As GPs are paid to record smoking status smoking behaviour is robustly recorded in the CPRD.^21^ Marston *et al*^21^ found that 84% of patients had smoking status recorded within a year of registering at a GP, and that smoking prevalence rates by age were similar in CPRD and the Health Survey of England. Booth *et al*^22^ found that the difference in prevalence of smoking estimate between the CPRD and the Health Survey for England was less than 1%, and the mean difference was 0.1% (95% CI −1.5% to 1.7%). Using unpublished data from CPRD sampled as part of the research reported in Thomas *et al*^13^ we found that 74% of patients prescribed smoking cessation medication had a subsequent record indicating smoking status. Of these 66% were indicated as current smokers and 33% as ex-smokers. We will initially define a patient as relapsed if they have any record indicating that the patient is a current smoker after their first prescription of a smoking cessation therapy. We will not be able to determine the smoking status of patients who do not return to the GP. Therefore, we will perform sensitivity analyses to examine whether the assumptions made about the smoking status of individuals who are not observed affect the results. For example, we will conduct a sensitivity analysis to see if the results are altered by assuming that patients with missing data have relapsed, or by assuming that patients with missing outcome data have achieved abstinence.

##### Outcome 2: Frequency of GP and hospital attendance

We will define service use as the number of visits to GP and hospitals in the 3, 6, 9, 12, 24 and 48 months after first prescription. We will define GP appointments using the clinical data file of the CPRD. This includes all the diagnoses and symptoms that GPs record about all of their patients. As with the other outcomes, the vast majority of diagnoses and symptoms include the date on which the data were added to the database. We will use these dates to calculate the number of times each patient attends primary care. We will define the hospital visits outcome using the linked HES data. We will investigate all-cause hospitalisation and three specific causes of hospitalisation: (1) diseases of respiratory system (ICD-10=J00-J99), (2) cardiovascular disease (ICD-10=I00-I52) and (3) anxiety and depression (ICD-10=F31.3, F31.4, F31.5, F32, F40-F48). Causes of hospitalisation are available for approximately half of the sample. Again these data contain the date on which the event occurred, which we will use to define attendance to secondary care within 3, 6, 9, 12, 24 and 48 months after first prescription.

##### Outcome 3: All-cause and cause-specific mortality

We will define all-cause and cause-specific mortality using the linked ONS mortality data set. These include the date of death and cause of death using ICD-9 codes. We will investigate three specific causes of mortality: (1) diseases of respiratory system (ICD-10=J00-J99), (2) cardiovascular disease (ICD-10=I00-I52), and (3) anxiety and depression (ICD-10=F31.3, F31.4, F31.5, F32, F40-F48). We will use validated code lists for each outcome.

##### Outcome 4: Incident respiratory illness, myocardial infarction, depression or anxiety

We will define the adverse event outcomes using the diagnosis records from the Clinical and Referral files in the CPRD. These files record all the diagnoses that the GPs input into their computer system. Each record in the table is given a diagnosis code based on the Read code categorisation. We will use validated Read code lists, for the three adverse event outcomes, respiratory illnesses, myocardial infarction or depression and anxiety, please see the cited papers for Read code lists.^23–25^ For eligible patients we will extract all records from the Clinical and Referral Tables that indicate the patient either received a specific diagnosis or were referred for a specific diagnosis. As with the therapy records for prescriptions described above, each Clinical and Referral Record indicates the date the information was inputted into the system. We will use this date to define the date that the diagnosis was performed. We will define a set of outcomes within 3, 6, 9, 12, 24 and 48 months after first prescription.

#### Confounding factors

We will include gender, age in years at time of first prescription, previous psychiatric illness/consultation, previous use of psychotropic medications such as hypnotics, antipsychotics and antidepressants, previous self-harm, measures of alcohol consumption where appropriate mean/median number of GP visits per year, body mass index, SEP (deprivation score for area or residence) and major chronic illness (including: diabetes, cancer, arthritis) using the Charlson index.^26^ ^27^ Relevant Read codes will be identified either by validated code lists or by searching for each of these events in the Read code dictionaries to identify any missing Read codes. Collider bias is a potential threat to the analysis; this type of bias occurs when the association between two variables changes on conditioning of a third variable if the third variable is affected by the first two variables. Collider bias could occur if we conditioned on events that happened as a result of the prescription the patient was issued. To prevent this bias from affecting our results, we will define each covariate using data inputted prior to the first prescription.^28^ If there are missing data in the covariates we will consider using multiple imputation.

##### Follow-up

Follow-up will end with the earliest of either an ‘event’ or censoring due to the end of registration or death.

### Statistical analysis

For investigating the effects of varenicline use on each outcome (long-term smoking cessation, frequency of GP and hospital attendance, all-cause and cause-specific mortality, primary care diagnosis of respiratory illness, myocardial infarction, depression or anxiety), we will report a conventional multivariable-adjusted Cox regression, propensity score regression and instrumental variable analysis.

#### Analysis 1: Conventional cox regression

In our first analysis, a conventional observational analysis, we will estimate HRs of the outcomes using Cox-proportional hazards models and the actual prescriptions issued to the patients.^29^ Each patient's date of entry into the cohort will be the date they were first prescribed a smoking cessation therapy. The date of exit for each outcome will be the date on which they first have an event, or are censored due to end of follow-up or death or leaving the practice. We will report these associations adjusted for basic confounders (age and gender), and results adjusted for all measured covariates described above.

#### Analysis 2: Propensity score regression

In our second analysis we will construct a sample of patients balanced on covariates and risk factors using a propensity score.^30–33^ We will construct propensity scores using a logistic regression of the actual treatment received on the covariates described above. Therefore, each participant's propensity score will be their conditional probability (odds) of receiving varenicline versus nicotine replacement therapy. We will match each patient receiving varenicline to another patient receiving nicotine replacement therapy with the closest propensity score on a ratio of 1:1 using a nearest neighbour algorithm with no replacement, and matching will be restricted to the common support region. Patients outside the common support region are those prescribed varenicline with propensity scores higher than any patient prescribed nicotine replacement therapy and vice-versa. We will estimate HRs of the outcomes using the propensity score matched sample using Cox regressions using the same entry and exit information as the conventional Cox regression analysis described above.

#### Analysis 3: Instrumental variable analysis

In our third analysis, we will estimate the effects of smoking cessation therapies on the outcomes using physicians’ prescribing preferences as instruments for the prescriptions the GPs issue to their patients. We cannot directly measure the physicians’ preferences; therefore, we will use the prescriptions they issued to their previous patients as a proxy for their preferences. For example, if the instrument was based on just one previous prescription, physicians who previously prescribed varenicline would be categorised as a varenicline prescriber. As with our previous studies we will use seven prior prescriptions to improve the strength of the instruments.^13^ ^34^ ^35^ Using multiple prior prescriptions will maximise power. We will report risk differences in the outcomes using additive structural mean models estimated via the generalised method of moments.^36–38^

We will categorise each of the adverse event outcomes as occurring within 3, 6, 9, 12, 24 and 48 months of first prescription. We will do this because methods for conducting survival analysis using instrumental variables are not well developed. We will use Stata 13.1 SE to generate all results. The instrumental variable analysis will be conducted using the ivreg2 command and psmatch2 will be used to construct the propensity score.^31^ ^39^ ^40^ All SEs will be estimated using cluster robust SEs, which account for clustering of patients within practices.

### Socioeconomic variation in effectiveness of smoking cessation treatments

This project will use the entire sample of patients indicated as a smoker at any point after 1 September 2006. We will assign a measure of area level deprivation to each patient using their home address postcode and to each GP using the GP postcode. Deprivation levels will be based on the Indices of Multiple Deprivation (IMD), which are available from the ONS and are updated every 2 years. We will use the most recent IMD statistics preceding the date of entry into the study for each patient. Although area level deprivation statistics will only be a proxy for individual level deprivation, these demonstrate the expected associations with smoking prevalence.^41^ We will investigate whether the proportion of smokers who attend their GP for smoking cessation treatment differs by IMD, and whether there are any differences in prescribing of varenicline versus nicotine replacement products between areas of high and low deprivation.

By using individual and GP level IMD codes, we will investigate whether the effects of smoking cessation therapies differ by IMD at the level of GPs and at the individual level. We will investigate treatment compliance by reporting the total number of prescriptions issued after the initial prescription. We will estimate the effects of smoking cessation therapies within subgroups defined by IMD level at the individual and GP level using the three methods described above, multivariable-adjusted Cox regression, propensity score regression and instrumental variable analysis.^29^ ^32^ ^42^ The cohort of patients will be defined as described above. We will report these associations adjusted for basic confounders (age and gender), and results adjusted for all measured covariates described above. Analyses will account for clustering of patients by GPs.

## Ethics approval, peer review, data curation and dissemination

Access to the CPRD data is governed by its Independent Scientific Advisory Committee (ISAC). The empirical research described in this proposal significantly expands on our existing work. We have received approval for this project protocol from ISAC (protocol number 15_107). We will comply with all requirements of ISAC requirements for publications based on CPRD data, for example, including the ISAC study protocol as an appendix to published papers. This protocol has been peer reviewed separately as part of the NIHR Health Technology Assessment board's efficient study designs call (proposal ID 14/49/94) and the ISAC expert advisory board. The data produced as part of this study will be made available via a system of managed open access—interested researchers who obtain necessary approvals from ISAC will be permitted access to the data generated during this study.

Key findings will be collated to form evidence-based recommendations which will be communicated to the FDA and the Medicines and Healthcare Products Regulatory Agency (MHRA), with the aim of improving the evidence base to inform advice to prescribers and patients. We will also aim to publish findings in peer-reviewed journals and present our work at national and international conferences.
